# Treatment consequence and adverse events of cyclin-dependent kinase 4/6 inhibitors on patients with hormone receptor-positive, HER2-negative metastatic breast cancer: a systematic review and meta-analysis

**DOI:** 10.1080/07853890.2025.2557509

**Published:** 2025-09-08

**Authors:** Hsiang-Ying Wu, Chia-Sung Chang, Wei-Hong Cheng, Jin-Hua Chen, Yuan-Hung Wang

**Affiliations:** a Graduate Institute of Clinical Medicine, College of Medicine, Taipei Medical University, Taipei, Taiwan; b School of Pharmacy, College of Pharmacy, Taipei Medical University, Taipei, Taiwan; c Division of Hematology and Oncology, Department of Internal Medicine, Shuang Ho Hospital, Taipei Medical University, New Taipei City, Taiwan; d Graduate Institute of Data Science, College of Management, Taipei Medical University, Taipei, Taiwan; e Office of Data Science, Taipei Medical University, Taipei, Taiwan; f Department of Medical Research, Shuang Ho Hospital, Taipei Medical University, New Taipei City, Taiwan

**Keywords:** Breast cancer, CDK4/6 inhibitor, endocrine therapy, meta-analysis

## Abstract

**Background:**

Although some studies have indicated that CDK4/6 inhibitors are beneficial for the progression-free survival (PFS) and overall survival (OS) in breast cancer, evidence regarding the assessment of clinical response remains insufficient. Therefore, this study aims not only to evaluate the efficacy and safety of CDK4/6 inhibitors combined with endocrine therapy in HR(+)/HER2(−) metastatic breast cancer, but also to analyze the objective response rate (ORR) and clinical benefit rate (CBR), providing comprehensive clinical outcome insights.

**Materials and methods:**

A literature search was performed in PubMed, Embase, Cochrane Library, and ClinicalTrials.gov focusing on studies published before 2022. The meta-analysis followed PRISMA guidelines and used RevMan 5.3 to conduct the analysis.

**Results:**

Eleven clinical trials published between 2015 and 2022 were included in our meta-analysis, with a total of 5572 eligible patients. This meta-analysis found that HR(+)/HER2(−) metastatic breast cancer treated with CDK4/6 inhibitors plus endocrine therapy can significantly improve progression-free survival(PFS) (HR: 0.55; *p* < 0.001), overall survival (OS) (HR: 0.79; *p* < 0.001), objective response rate (RR = 1.50; *p* < 0.001), clinical benefit rate (RR = 1.18; *p* < 0.001) and decrease progressive disease rate (RR = 0.49; *p* < 0.001). Clinicians need to be aware of hematological toxicities, abnormal liver function, and venous thromboembolism in the use of CDK4/6 inhibitors. Furthermore, the combination regimen also showed longer PFS in subgroup analysis. However, Asians, the number of metastasis sites, and patients using letrozole subgroups did not demonstrate differences in OS between the combination regimen and endocrine therapy alone.

**Conclusion:**

This meta-analysis highlights the improvement of PFS, OS, ORR, and CBR in HR(+)/HER2(−) metastatic breast cancer for CDK4/6 inhibitors, with manageable and reversible toxicities. Clinicians should be aware of hematological toxicities, liver function abnormalities, and venous thromboembolism when using CDK4/6 inhibitors. These findings make CDK4/6 inhibitors a pivotal treatment option.

## KEY MESSAGES

CDK4/6 inhibitor combined with endocrine therapy can prolong progression-free survival, overall survival, objective response rate, and clinical benefit rate, and decrease progressive disease rate.

Hematological toxicities are the main concern for CDK4/6 inhibitors. Clinicians still need to be aware of adverse events such as abnormal liver function and venous thromboembolism.

CDK4/6 inhibitors plus endocrine therapy improved PFS in all subgroups, but no difference in OS was observed in Asians, the number of metastasis sites, and patients using letrozole compared to endocrine therapy alone.

CDK4/6 inhibitors plus endocrine therapy is a pivotal treatment option for HR(+)/HER2(−) breast cancer based on their efficacy and safety.

## Introduction

Cancer is one of the major global health issues. In particular, breast cancer has been the most common cancer among women and is one of the leading causes of cancer-related deaths. Since mid-2000s, the incidence of breast cancer in women has been increasing by approximately 0.5% annually [1]. It is estimated that by 2040, there will be more than 3 million new cases of breast cancer and over 1 million breast cancer-related deaths globally each year [2]. Given the growing burden of this disease, research into breast cancer is of great importance to provide evidence and ultimately improve treatment strategies.

Breast cancer can be classified into molecular subtypes using immunohistochemistry, which allows for more targeted treatment strategies. The main subtypes include hormone receptor(HR)-positive, human epidermal growth factor receptor 2(HER2)-enriched, and triple-negative breast cancer [3]. HR-positive breast cancer is characterized by the presence of either estrogen receptor (ER) or progesterone receptor (PR) positive. Given the diversity of breast cancer subtypes, treatment approaches vary accordingly. According to NCCN guidelines, treatment options include surgery, radiation therapy and drug therapy [4]. Beyond traditional chemotherapy and endocrine therapy, advancements in molecular biology have led to the development of immunotherapy (e.g. PD-1/PD-L1 inhibitors) and targeted therapies (e.g. CDK4/6 inhibitors and PARP inhibitors) [5–7].

Cyclin-dependent kinase (CDK) and checkpoints are essential in cell cycle. However, CDK4/6 inhibitors such as palbociclib, ribociclib, and abemaciclib block the phosphorylation of retinoblastoma (RB) protein and prevent the release of E2F transcription factor. This CDK-RB-E2F transcription factor pathway halts the transition of G1 to S phase and disturbs DNA synthesis [8–11]. Recently, various clinical trials have discussed the efficacy and safety of CDK4/6 inhibitors in patients with breast cancer, especially in HR(+)/HER2(−) breast cancer.

Several clinical trials have demonstrated that CDK4/6 inhibitors combined with endocrine therapy can extend progression-free survival (PFS) of HR(+)/HER2(−) metastatic breast cancer. However, overall survival (OS) has not met expectations, and objective response rates (ORR) have different results in the research. Additionally, the impact of various confounding factors on a patient’s prognosis remains unresolved in clinical practice. Furthermore, most meta-analyses primarily include data from older trials such as PALOMA, MONALESSA, and MONARCH, which limits the inclusion of novel clinical trial data and recent clinical evidence.

Therefore, this systematic review and meta-analysis aims to provide a comprehensive and reliable evaluation of the efficacy and safety of CDK4/6 inhibitors combined with endocrine therapy (ET) on the prognosis of patients with HR(+)/HER2(−) metastatic breast cancer. Additionally, this study seeks to incorporate more clinical trial data and assess the impact of a wider range of confounding factors.

## Materials and methods

### Search strategy

A search for relevant research that investigates the efficacy and safety of CDK4/6i in HR(+)/HER2(−) breast cancer patients was conducted in PubMed, Embase, Cochrane Library, and ClinicalTrials.gov. The search period was developed before 2022. Keywords used in the search were as follows to find the relevant clinical trials: (‘breast cancer’ OR ‘metastatic breast cancer’ OR ‘advanced breast cancer’) and (‘CDK4/6 inhibitor’ OR ‘abemaciclib’ OR ‘ribociclib’ OR ‘palbociclib’) and (‘progression-free survival’ OR ‘overall survival’). This study has been registered in PROSPERO system (Registration number: CRD42022346455).

### Selection criteria

To select studies that align with the research question, a set of standardized selection criteria was required, as outlined below.

Inclusion criteria: 1-All studies are published in English; 2-Second or third-phase clinical trials involve HR(+)/HER2(−-) metastatic breast cancer patients in either first-line or second-line treatment; 3-Both single-center and multi-center studies, as well as open-label and double-blind trials, are included, with no restrictions on sample size.

Exclusion criteria: 1 – Phase I clinical trials, meta-analyses, systematic review, preclinical studies, observational studies, single-arm trials, non-randomized studies, and case reports were excluded; 2 – Studies related to early-stage breast cancer, neoadjuvant or adjuvant therapy, triple-negative breast cancer, HER2(+) breast cancer; 3 – Control groups treated with chemotherapy, immunotherapy, or CDK4/6 inhibitors, and cases where the experimental group did not combine endocrine therapy with CDK4/6 inhibitors were excluded.

### Data extraction and quality assessment

The extracted data from the literature included medium follow-up time, hazard ratio (HR), and 95% confidence intervals (CI) from to measure progression-free survival (PFS) and overall survival (OS). Additionally, odds ratios (OR) were used to evaluate objective response rate (ORR) and clinical benefit rate (CBR). Key patient-specific information such as disease progression, treatment lines, metastatic sites, race, hormone receptor status, age, ECOG, chemotherapy usage, number of metastatic sites, menopausal status, status of endocrine therapy sensitivity or resistance, and endocrine therapy medications will be systematically analyzed along with common adverse events to provide a comprehensive evaluation. For quality assessment, Cochrane’s Risk of Bias Tool was used to assess the quality of the included studies.

### Statistical analysis

Review Manager 5.3 was used to perform the meta-analysis. Hazard ratios (HR) were applied to analyze the continuous data, while Risk ratios (RR) were used for categorical data. Results were reported with 95% confidence intervals (CIs), and p-values below 0.05 were considered statistically significant. Heterogeneity was assessed using Cochrane’s *Q* statistics and *I*^2^ statistics. The *p*-value of the Cochrane’s Q test was less than 0.1, which was considered statistically significant. The *I*^2^ values were classified as follows: <25% indicated low heterogeneity, 25%–75% indicated moderate heterogeneity, and >75% indicated high heterogeneity. For sensitivity analysis, a dichotomous analysis was conducted to assess tumor response to treatment (based on RECIST 1.1) and adverse effects, with results presented in RR and their 95% CI using Mantel–Haenszel test. A random-effects model was applied for all analyses.

## Result

### Literature search

**Figure 1.**
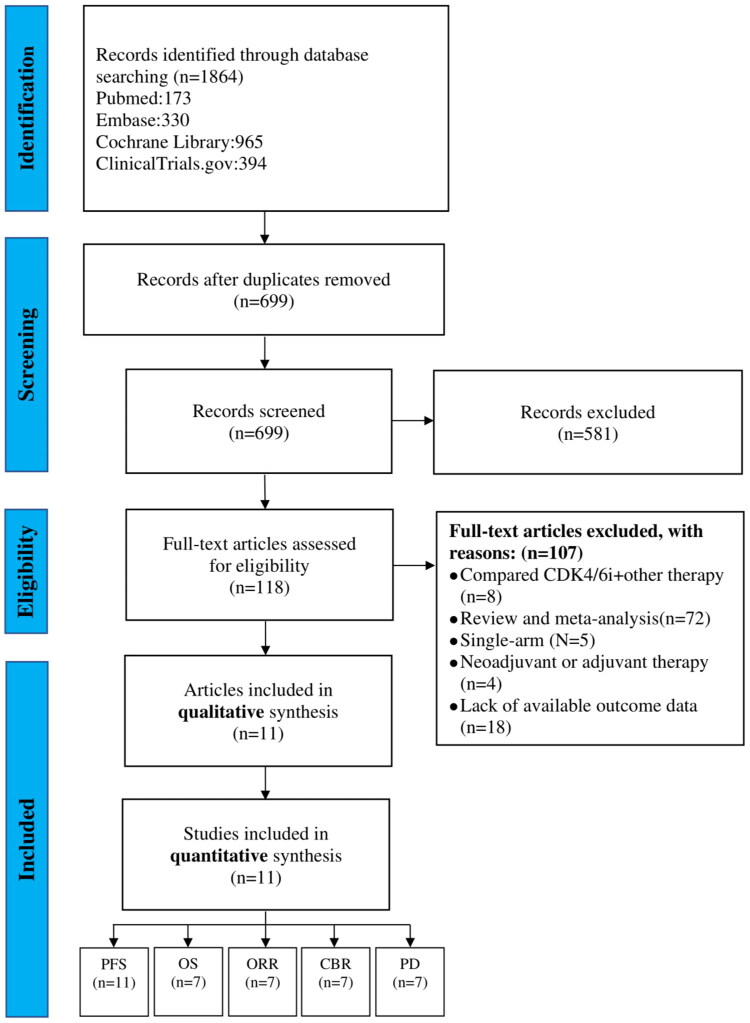
The PRISMA flowchart of literature searching strategy for this systematic review and meta-analysis. CDK4/6i-cyclin-dependent 4 and 6 inhibitors; PFS-progression-free survival; OS-overall survival; ORR-objective response rate; CBR-clinical benefit rate; PD-progressive disease.

A comprehensive search of electronic databases identified a total of 1,864 relevant article, 11 studies involving 5,572 patients were eligible for this meta-analysis (Figure 1). Of these, 3,372 patients received CDK4/6i + ET, while 2196 received et al.one. In these 11 studies, CDK4/6i was used as a first-line treatment for metastatic breast cancer in seven studies (PALOMA-1/2/4, MONALEESA-2/7, MONARCH-3/Plus cohort A) [12–18], as a second-line treatment in 4 studies (PALOMA-3, FLIPPER, MONARCH-2/Plus cohort B) [18–21], and in either first- or second-line treatment in 1 study (MONALEESA-3) [22]. The characteristics of each randomized clinical trial are summarized in Table 1.

**Table 1.** Baseline characteristics of randomized control trials (RCT) in the meta-analysis.

Clinical trial	Line	Phase	Patients (Experimental: Control)	Blinded experiment	Menopause	Experimental Arm	Control Arm	PFS			OS		
								Months	HR (95% CI)	P value	Months	HR (95% CI)	*p*-value
PALOMA-1 Finn,2015	1st	II	165 (84:81)	open-label	post-menopausal	Palbociclib + letrozole	letrozole	20.2 vs. 10.2	0.488 (0.319–0.748)	<0.001	37.5 vs. 34.5	0.897 (0.623–1.294)	0.281
PALOMA-2 Finn,2016	1st	III	666 (444:222)	double-blind	post-menopausal	Palbociclib + letrozole	letrozole (+ GnRHa in pre/peri pts)	27.6 vs. 14.5	0.58 (0.46–0.72)	<0.001	53.9 vs. 51.2	0.956 (0.777–1.177)	0.3378
PALOMA-3 Verma,2016	2nd	III	521 (347:174)	double-blind	pre/post-menopausal	Palbociclib + Fulvestrant	placebo + Fulvestrant	12.9 vs. 5.5	0.49 (0.37–0.65)	<0.001	34.9 vs. 28.0	0.85 (0.64–1.13)	0.09
PALOMA-4 Xu,2022	1st	III	340 (169:171)	double-blind	post-menopausal	Palbociclib + letrozole	letrozole	21.5 vs. 13.9	0.68 (0.53–0.87)	0.0012	NA	NA	NA
FLIPPER Albanell,2021	2nd	II	189 (94:95)	double-blind	post-menopausal	Palbociclib + Fulvestrant	placebo + Fulvestrant	31.8 vs. 22.0	0.48 (0.37–0.64)	0.001	NA	NA	NA
MONALEESA-2 Hortobagyi,2016	1st	III	668 (334:334)	double-blind	post-menopausal	Ribociclib + letrozole	letrozole	25.3 vs. 16.0	0.568 (0.457–0.704)	<0.001	63.9 vs. 51.4	0.76 (0.63–0.93)	0.008
MONALEESA-3 Slamon,2018	1st + 2nd	III	726 (484:242)	double-blind	post-menopausal	Ribociclib + Fulvestrant	Fulvestrant	23.5 vs. 12.8	0.593 (0.480–0.732)	<0.01	53.7 vs. 41.5	0.73 (0.59–0.90)	0.00455
MONALEESA-7 Tripathy,2018	1st	III	672 (335:337)	double-blind	pre/peri-menopausal	Ribociclib + T/AI+OFS	T/AI+OFS	23.8 vs. 13.0	0.55 (0.44–0.69)	<0.001	58.7 vs. 48.0	0.76 (0.61–0.96)	0.00973
MONARCH plus (Cohort A) Zhang,2020	1st	III	306 (207:99)	double-blind	post-menopausal	Abemaciclib + NSAI	placebo + NSAI	NR vs. 14.7	0.499 (0.346–0.719)	<0.001	NA	NA	NA
MONARCH plus (Cohort B) Zhang,2020	2nd	III	157 (104:53)	double-blind	post-menopausal	Abemaciclib + Fulvestrant	placebo + Fulvestrant	11.5 vs. 5.6	0.376 (0.240–0.588)	<0.001	NA	NA	NA
MONARCH-2 Sledge,2017	2nd	III	669 (446:223)	double-blind	pre/peri-menopausal	Abemaciclib + Fulvestrant	Fulvestrant	16.4 vs. 9.3	0.553 (0.449–0.681)	<0.01	46.7 vs. 37.3	0.757 (0.606–0.945)	0.01
MONARCH-3 Goetz,2017	1st	III	493 (328:165)	double-blind	post-menopausal	Abemaciclib + NSAI	NSAI (+ GnRHa in pre/peri pts)	29.0 vs. 14.8	0.518 (0.415–0.648)	<0.001	67.1 vs. 54.5	0.754 (0.584–0.974)	0.0301

NSAI-Non-steroidal aromatase inhibitor; GnRHa-Gonadotropin-releasing hormone agonist; T/AI-Tamoxifen/Aromatase inhibitor; OFS-Ovarian function suppression; pre/peri- pre/peri-menopausal; pts-patients; NA-Not available.

### Primary outcome

#### Analysis of PFS and OS

**Figure 2.**
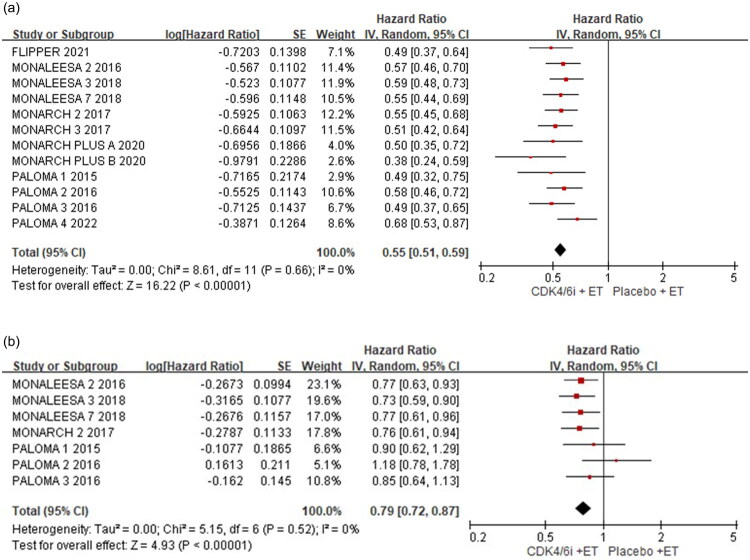
Forest plot of progression-free survival (PFS) and overall survival(OS) in combination treatment versus endocrine therapy alone for primary outcome (A) progression-free survival (B) overall survival.

Of these 11 studies, the result demonstrated that CDK4/6i + ET significantly improved PFS compared to control group (HR = 0.55; 95% CI: 0.51–0.59; *p* < 0.001) with no heterogeneity observed (I^2^=0%; *p* = 0.66) (Figure 2a). Seven studies, involving 2,474 patients in experimental group and 1,613 in control group, reported OS, including PALOMA-1/2/3 [12,13,19], MONALEESA-2/3/7 [15,16,22], and MONARCH-2 [21]. The findings indicated that CDK4/6i + ET have significantly longer OS compared to control group (HR= 0.79; 95% CI: 0.72–0.87; *p* < 0.001) with no significant heterogeneity (*I*^2^=0%; *p* = 0.52) (Figure 2b).

### Secondary outcome

#### Analysis of objective response rate and clinical benefit rate

Eleven studies were included in this analysis. Overall response rate (ORR) comprises two sub-outcomes: complete response (CR) and partial response (PR). The result indicated that the group receiving CDK4/6i + ET shows a higher ORR compared to control group. In Intention-to-Treat (ITT) analysis, risk ratio (RR) was 1.50 (95% CI: 1.33–1.70; *p* < 0.001), with similar findings observed in patients with measurable disease analysis. Moderate heterogeneity was noted in ITT (*I*^2^=53%; *p* = 0.02) and measurable disease analysis (I^2^=58%; *p* = 0.008).

Regarding clinical benefit rate (CBR), which includes CR and PR with lasting stable disease (SD) for at least six months, patients receiving combined therapy demonstrated better outcomes. In ITT analysis, the RR was 1.18 (95% CI: 1.09–1.27; *p* < 0.001), with consistent results in patients with measurable disease. However, substantial heterogeneity was observed in ITT (*I*^2^=74%; *p* < 0.001) and measurable disease (*I*^2^=65%; *p* = 0.002) (Table 2).

**Table 2.** Summary of objective response rate (ORR) and clinical benefit rate(CBR) in intention-to-treat(ITT) and measurable disease analysis.

	Palbociclib		Ribociclib		Abemaciclib		Overall	
	Palbociclib plus ET	ET alone	Ribociclib plus ET	ET alone	Abemaciclib plus ET	ET alone	CDK4/6i plus ET	ET alone
Best overall response, n (%)	1138 (60)	743 (40)	1153 (56)	913 (44)	1085 (67)	540 (33)	3376 (61)	2196 (39)
Objective response rate (ORR), n (%)								
CR + PR	395 (66)	203 (34)	430 (64)	244 (36)	470 (79)	127 (21)	1295 (69)	574 (31)
Risk ratio (95% CI)	1.32(1.12–1.56)		1.44(1.27–1.65)		1.94(1.38–2.73)		1.50(1.33–1.70)	
P-value	0.001		<0.001		<0.001		<0.001	
Clinical benefit rate (CBR), n (%)								
CR + PR+SD ≥24 weeks	895 (65)	485 (35)	871 (58)	630 (42)	563 (71)	233 (29)	2329 (63)	1348 (37)
Risk ratio (95% CI)	1.24(1.05–1.46)		1.11(1.06–1.18)		1.24(1.02–1.51)		1.18(1.09–1.27)	
P-value	0.009		<0.001		0.03		<0.001	
Patients with measurable disease	878 (60)	581 (40)	904 (56)	701(44)	841 (67)	417 (33)	2623 (61)	1699 (39)
Objective response rate (ORR), n (%)								
CR + PR	395 (67)	198 (33)	427 (64)	243 (36)	467 (79)	126 (21)	1289 (69)	567 (31)
Risk ratio (95% CI)	1.39(1.15–1.68)		1.41(1.25–1.60)		1.94(1.37–2.77)		1.51(1.33–1.71)	
P-value	<0.001		<0.001		<0.001		<0.001	
Clinical benefit rate (CBR), n (%)								
CR + PR+SD ≥24 weeks	580 (60)	381 (40)	683 (59)	469 (41)	457 (72)	177 (28)	1720 (63)	1027 (37)
Risk ratio (95% CI)	1.21(1.04–1.41)		1.15(1.08–1.23)		1.29(1.07–1.54)		1.19(1.11–1.28)	
P-value	0.01		<0.001		0.006		<0.001	

CR-complete response; PR-partial response; SD-stable disease; CDK4/6i-cyclin-dependent kinase 4 and 6 inhibitors; ET-endocrine therapy.

#### Analysis of progressive disease

This analysis confirmed that CDK4/6i + ET effectively slows disease progression. In ITT and measurable disease analyses, the RR is 0.49 (95% CI: 0.42–0.56; *p* < 0.001) and 0.49 (95% CI: 0.41–0.58; *p* < 0.001), respectively (Table 3). When comparing treatment outcomes across clinical trials, the results are nearly identical. However, the risk ratio in ITT and measurable disease analysis in PALOMA-4 [14] is 1.01 (95% CI: 0.55–1.88) and 0.49 (95% CI: 0.65–2.54), respectively, and lacks statistical significance.

**Table 3.** Summary of progressive disease in Intention-to-treat(ITT) and measurable disease analysis.

	Palbociclib		Ribociclib		Abemaciclib		Overall	
	Palbociclib plus ET	ET alone	Ribociclib plus ET	ET alone	Abemaciclib plus ET	ET alone	CDK4/6i plus ET	ET alone
Best overall response, n (%)	1138 (60)	743 (40)	1153 (56)	913 (44)	1085 (67)	540 (33)	3376 (61)	2196 (39)
Progressive disease	117 (46)	140 (54)	91 (41)	132 (59)	72 (46)	85 (54)	280 (44)	357 (56)
Risk ratio (95% CI)	0.50 (0.40–0.63)		0.52 (0.40–0.67)		0.42 (0.31–0.57)		0.49(0.41–0.58)	
P-value	<0.001		<0.001		<0.001		<0.001	
Patients with measurable disease	815 (61)	517 (39)	904 (56)	701 (44)	574 (67)	279 (33)	2293 (61)	1497 (37)
Progressive disease	96 (47)	109 (53)	73 (40)	110 (60)	57 (47)	64 (53)	226 (44)	283 (56)
Risk ratio (95% CI)	0.53 (0.41–0.67)		0.48 (0.36–0.64)		0.44 (0.30–0.64)		0.50(0.41–0.60)	
P-value	<0.001		<0.001		<0.001		<0.001	

CDK4/6i-cyclin-dependent kinase 4 and 6 inhibitors; ET-endocrine therapy.

#### Analysis of adverse effects

In clinical trials, the common adverse effects of palbociclib and ribociclib are neutropenia, whereas for abemaciclib, it is diarrhea. All three CDK4/6i can induce grade three to four neutropenia. In this analysis, the most frequent grade three to four adverse effect was pancytopenia, including the following: Neutropenia (RR = 26.87; 95% CI: 9.63–74.97; *p* < 0.001), Leukopenia (RR = 2.79; 95% CI: 1.34–5.87; *p* = 0.006), and Anemia (RR = 29.17; 95% CI: 9.10–93.48; *p* < 0.001). Abnormal liver function (RR = 4.19; 95% CI: 2.79–6.28; *p* < 0.001) was also the common grade three to four adverse effects of CDK4/6i. Among all trial participants, the addition of CDK4/6i significantly increases the risk of venous thromboembolism (RR = 2.65; 95% CI: 1.40–5.00; *p* = 0.03) (Table 4, Supplementary Figure 1a–n).

**Table 4.** Overall adverse events with all grades and grade 3 to 4.

	Ribociclib		Palbociclib		Abemaciclib		Total			
Treatment	CDK4/6i + ET (event/total)	Placebo + ET (event/total)	CDK4/6i + ET (event/total)	Placebo + ET (event/total)	CDK4/6i + ET (event/total)	Placebo + ET (event/total)	CDK4/6i + ET (event/total)	Placebo + ET (event/total)	Risk ratio (95%CI)	P value
All Grade										
Neutropenia	675/1153	24/913	961/1138	75/743	602/1085	42/540	2238/3376	141/2196	10.78(6.01–19.34)	<0.001
Nausea	430/1153	228/913	335/1138	157/743	417/1085	116/540	1182/3376	501/2196	1.54(1.35–1.76)	<0.001
Fatigue	341/1153	262/913	430/1138	189/743	404/1085	148/540	1175/3376	599/2196	1.27(1.02–1.59)	0.03
Diarrhea	325/1153	185/913	267/1138	113/743	896/1085	131/540	1488/3376	429/2196	1.94(1.07–3.51)	<0.001
Alopecia	264/1153	101/913	264/1138	87/743	163/1085	21/540	691/3376	209/2196	2.36(1.74–3.21)	<0.001
Vomiting	291/1153	138/913	169/1138	83/743	272/1085	63/540	732/3376	284/2196	1.68(1.31–2.15)	<0.01
Arthralgia	307/1153	251/913	276/1138	177/743	144/1085	82/540	727/3376	510/2196	0.97(0.88–1.07)	0.55
Leukopenia	352/1153	36/913	648/1138	62/743	456/1085	47/540	1456/3376	145/2196	6.31(4.83–8.24)	<0.001
Anemia	215/1153	62/913	421/1138	70/743	446/1085	46/540	1082/3376	178/2196	3.73(2.73–5.08)	<0.001
Decreased appetite	170/1153	108/913	154/1138	52/743	278/1085	62/540	602/3376	222/2196	1.74(1.20–2.53)	0.004
Thrombocytopenia	67/1153	11/913	305/1138	25/743	252/1085	23/540	624/3376	59/2196	6.23(4.63–8.39)	<0.001
Abnormal LFTse	318/1153	102/913	398/1138	210/743	400/1085	109/540	1116/3376	421/2196	1.76(1.17–2.65)	0.006
QTc prolong	68/1153	19/913	27/1138	14/743	0/1185	0/540	–	–	–	–
Grade 3–4										
Neutropenia	659/1153	15/913	783/1138	9/743	285/1085	14/540	1727/3376	38/2196	26.87(9.63–74.97)	<0.001
Nausea	17/1153	5/913	6/1138	7/743	17/1085	8/540	40/3376	20/2196	1.19(0.51–2.75)	0.69
Fatigue	20/1153	4/913	26/1138	4/743	25/1085	3/540	71/3376	11/2196	4.11(2.18–7.76)	<0.001
Diarrhea	12/1153	6/913	10/1138	5/743	105/1085	4/540	127/3376	15/2196	3.02(0.62–14.72)	0.17
Alopecia	0/1153	0/913	0/1138	0/743	0/1085	0/540	0/3376	0/2196	Not estimable	Not estimable
Vomiting	24/1153	5/913	4/1138	5/743	13/1085	8/540	41/3376	18/2196	1.22(0.37–4.01)	0.74
Arthralgia	9/1153	7/913	7/1138	6/743	4/1085	1/540	20/3376	14/2196	0.97(0.48–1.93)	0.93
Leukopenia	186/1153	6/913	344/1138	2/743	124/1085	5/540	654/3376	13/2196	29.17(9.10–93.48)	<0.001
Anemia	29/1153	16/913	56/1138	12/743	93/1085	9/540	178/3376	37/2196	2.79(1.34–5.81)	0.006
Decreased appetite	8/1153	1/913	8/1138	1/743	9/1085	3/540	25/3376	5/2196	2.7091.02-7.16)	0.05
Thrombocytopenia	6/1153	2/913	32/1138	1/743	29/1085	4/540	67/3376	7/2196	4.75(1.52–14.88)	0.007
Abnormal LFTse	87/1153	17/913	36/1138	8/743	82/1085	6/540	205/3376	31/2196	4.19(2.79–6.28)	<0.001
QTc prolong	2/1153	1/913	2/1138	0/743	0/1085	0/540	–	–	–	–
Serious Adverse Events										
*VTE	16/1153	4/913	11/1138	3/743	22/1085	2/540	49/3282	9/2101	2.65(1.40–5.00)	0.003

Abbreviations: CDK4/6i-cycline-dependant kinase 4 and 6 inhibitors; ET-endocrine therapy; Abnormal LFTse-abnormal liver function tests elevation; VTE-venous thromboembolism.

#### Subgroup analysis

In this section, we conducted twelve subgroup analyses to evaluate the effects of various subgroups on PFS and OS. Experimental group consisted of patients receiving CDK4/6i + ET, while control group received either placebo or endocrine therapy alone. For PFS, a significant trend in favor of using CDK4/6i combined therapy was observed across most subgroups. However, for OS, no significant differences were noted in subgroups of Asians, the number of metastasis sites, or patients using letrozole. A detailed summary of the results is provided in Table 5 (Supplementary Figure 2a–m).

**Table 5.** Subgroup analysis of progression-free survival (PFS) and overall survival(OS).

	CDK4/6 inhibitor + Endocrine therapy vs. Placebo + Endocrine therapy			
	PFS		OS	
Subgroup	Hazard Ratio	95% CI	Hazard Ratio	95% CI
Treatment lines				
First line	0.57	0.51-0.63	0.8	0.69-0.93
Second line	0.52	0.45-0.60	0.77	0.67-0.89
Metastases (Visceral)				
Visceral metastases	0.56	0.51-0.62	0.81	0.72-0.91
Non-visceral metastases	0.53	0.45-0.61	0.78	0.68-0.90
Metastases (Bone)				
Bone metastases	0.54	0.41-0.70	0.73	0.60-0.88
Non-bone metastases	0.58	0.52-0.65	0.83	0.79-0.98
Race				
Asian	0.48	0.39-0.60	0.76	0.56-1.02
Non-Asian	0.58	0.51-0.67	0.82	0.74-0.91
Hormone receptor status				
ER and PR positive	0.53	0.47-0.60	0.77	0.68-0.89
Other	0.52	0.42-0.65	0.71	0.56-0.89
Age group				
<65 years old	0.53	0.48-0.58	0.82	0.71-0.93
≧65 years old	0.58	0.50-0.68	0.8	0.69-0.94
ECOG				
0	0.57	0.51-0.63	0.79	0.65-0.96
≧1	0.51	0.47-0.62	0.79	0.68-0.91
Chemotherapy usage				
For (neo)adjuvant therapy	0.56	0.47-0.66	0.82	0.71-0.93
No prior chemotherapy	0.46	0.36-0.59	0.8	0.66-0.97
Number of Metastatic sites				
1	0.55	0.46-0.67	0.86	0.65-1.12
2	0.44	0.36-0.54	0.74	0.49-1.13
≧3	0.56	0.49-0.63	0.79	0.66-0.94
Menopausal status				
Post-menopausal	0.55	0.51-0.60	0.8	0.73-0.88
Pre/Peri-menopausal	0.52	0.43-0.63	0.76	0.60-0.96
Endocrine therapy status				
No prior ET	0.55	0.46-0.66	–	–
Endocrine sensitive	0.56	0.50-0.62	0.82	0.71-0.96
Endocrine resistance	0.51	0.45-0.58	0.77	0.67-0.89
Primary endocrine resistance	0.49	0.36-0.67	–	–
Secondary endocrine resistance	0.54	0.44-0.65	–	–
Endocrine Therapy Medication				
Letrozole	0.57	0.51-0.62	0.86	0.72-1.02
Fulvestrant	0.53	0.47-0.59	0.77	0.67-0.88

Abbreviation: ECOG: eastern cooperative oncology group performance test.

#### Quality assessment

**Figure 3.**
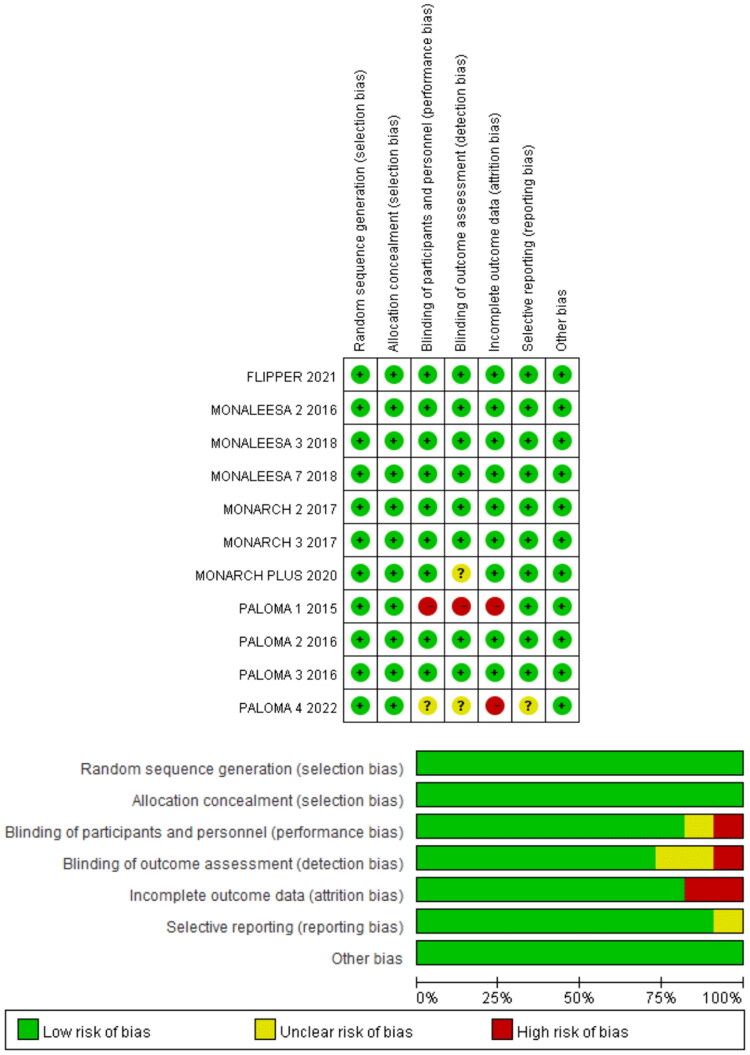
Risk of bias and quality assessment of included clinical trials.

Except for PALOMA-1 [12], an open-label trial, all other studies were multicenter, randomized, double-blinded, and placebo-controlled, demonstrating low selection bias. However, PALOMA-4[14] lacked sufficient information regarding whether the clinical trial investigators informed participants how outcomes would be measured and interpreted. Additionally, it did not provide complete data for the subgroups of interest. Detailed information on quality assessment is presented in Figure 3.

#### Sensitivity analysis

MONALEESA-3 [22] revealed high heterogeneity between CDK4/6i combined therapy and et al. one in PFS and OS of both Asian and non-Asian groups. This heterogeneity may be attributed to insufficient data in Asian group (Supplementary Table 1).

#### Publication bias

In this study, funnel plots for PFS, OS (Supplementary Figures 3 and 4), ORR and CBR (Supplementary Figures 5 and 6) are asymmetrical. This asymmetry is likely influenced by both small-study effects and differences in the treatment outcomes. However, tests for funnel plot asymmetry should be used only when there are more than 10 studies included in the meta-analysis, and we included 11 studies which are slightly larger than 10 [23]. Therefore, publication bias should be interpreted with caution.

## Discussion

CDK4/6 inhibitors have revolutionized the treatment landscape for HR(+)/HER2(−) metastatic breast cancer. Regulation agencies have approved them in combination with aromatase inhibitors or Fulvestrant. Although there is a broad consensus on their efficacy and safety, controversies remain in interpreting clinical trial data, especially in subgroup analysis [24]. To deliver comprehensive clinical insights and enhance clarity, this meta-analysis assesses the efficacy and safety profiles of CDK4/6 inhibitors, integrating ORR, CBR, and subgroup analysis to address treatment outcomes and variations across patient subgroups.

This meta-analysis confirms that CDK4/6 inhibitors significantly improve PFS and OS. PFS outcomes from PALOMA-1, PALOMA-2 and PALOMA-3 [12,13,19] demonstrated better efficacy of palbociclib plus letrozole than et al. one. However, their OS outcomes did not show significant efficacy. It might be too early to assess a difference in OS given the limited follow-up period. Additionally, there are other treatment options available for patients with HR(+)/HER2(−) metastatic breast cancer following endocrine therapy in combination with CDK4/6 inhibitors.

In terms of secondary outcomes, this study further reinforces that ORR and CBR associated with three CDK4/6i + ET demonstrate superior clinical efficacy, both in ITT and measurable disease analysis. Furthermore, the progressive disease rate exhibits positive outcomes, suggesting that CDK4/6i + ET effectively mitigates disease progression. Nonetheless, CBR and progressive disease rate analysis in PALOMA-4 failed to achieve statistical significance, presenting findings that contrast with the results of this meta-analysis. This discrepancy may be attributed to the limited sample size and the homogeneous racial composition of participants in PALOMA-4, which was restricted to an exclusively Asian cohort [14].

Despite CDK4/6i sharing a common mechanism targeting CDK-RB-E2F transcription factor pathway, they differ in CDK/cyclin target spectrum [25]. Therefore, CDK4/6i exhibit differences in both hematologic and non-hematologic toxicity profiles.

Hematologic toxicities are more common with palbociclib and ribociclib, especially grade 3 to 4 leukopenia and anemia. Abemaciclib also causes hematologic adverse effects but less frequently at high grades. Notably, these toxicities are reversible. Upon discontinuation of therapy, blood counts typically recover rapidly, distinguishing CDK4/6i induced myelosuppression from that caused by traditional chemotherapy [26].

Non-hematologic toxicities vary across agents. Abemaciclib is more often associated with gastrointestinal events, mainly low-grade diarrhea manageable with antidiarrheal medications or dose adjustments [27]. Cardiac and hepatic safety profiles also vary among agents. QT prolongation is the most frequently reported with ribociclib, followed by palbociclib, which showed a 14.8% incidence in the PALOMA-4 trial [14]. Abemaciclib has not been associated with QT prolongation in clinical studies, indicating a more favorable cardiac safety profile. All three agents can lead to liver enzyme elevations. For all-grade events, abemaciclib (36.9%) and palbociclib (35%) show higher incidence rates than ribociclib (27.6%). However, for grade 3 to 4 liver enzyme elevations, ribociclib (7.5%) and abemaciclib (7.6%) exceed palbociclib (3.1%), highlighting the importance of monitoring liver function during treatment.

Venous thromboembolism (VTE) is a known concern with CDK4/6i, particularly when combined with endocrine therapy [28,29]. Reported VTE incidences vary 4.7% for palbociclib in PALOMA-1; 2.3% and 3.4% for abemaciclib in MONARCH-2 and MONARCH-3, and 1.3% and 0.9% for ribociclib in MONALESSA-7 and MONALESSA-3, respectively [28,30–32]. Notably, this study observed the highest VTE incidence with abemaciclib and the lowest with palbociclib. These differences may reflect variations in study populations, sample sizes, or methods of data aggregation. In addition, the type of endocrine backbone may influence VTE risk. Tamoxifen is associated with a higher thromboembolic risk compared to aromatase inhibitors, and Fulvestrant may carry a slightly lower risk among endocrine agents [28]. Therefore, the choice of endocrine partner may influence the thromboembolic profile combined with CDK4/6 inhibitors.

In subgroup analysis of PFS, CDK4/6i + ET demonstrates significant benefits across all subgroups. However, trials such as FLIPPER [20] and MONALEESA-3 [22] show discrepancies, without PFS improvement observed in bone metastases and Asian subgroups, respectively. MONARCH PLUS [18] found no benefit in non-Asian subgroup, while a similar outcome was observed in both FLIPPER [20] and MONARCH PLUS [18] for either ER or PR-positive subgroup. These inconsistencies may stem from limited sample sizes or the percentage of hormone receptor positivity. Research indicated that tumors with less than 6% estrogen receptor (ER) positivity resemble the characteristics of triple-negative breast cancer. In addition, the prognosis may vary based on the positivity of progesterone receptor (PR), which suggests the importance of hormone therapy patterns in predicting treatment outcomes [33–35]. For the number of metastatic site subgroups, FLIPPER [20] reported no PFS improvement in patients with single-site metastasis. However, the trial did not provide detailed information about the metastatic organs involved. Other related studies [36–38] have shown that breast cancer with liver or brain metastasis tends to have worse prognosis compared to cases with bone or lung metastasis.

Regarding OS, this meta-analysis found significant improvements in most of the subgroups, though Asians and those with one, two metastatic sites, and patients using letrozole show no preference for either CDK4/6i + ET or et al. one. Trials like PALOMA-2 [13] indicated no evidence of significantly prolonged OS in the following subgroups: first-line therapy, non-bone metastases, patients under 65 years of age, those with an ECOG score of 0, individuals with no prior chemotherapy, and patients with metastases to more than three organs. The potential reasons for this phenomenon might include the higher mortality rates in CDK4/6i + ET group and high attrition rate of trial participants. The insignificant benefit observed in the Asian subgroup may be attributed to variations in the proportion of the Asian population across clinical trials and an insufficient Asian population size, which influence the generation of robust evidence. Additionally, various gene mutation profiles or cultural influences, such as dietary patterns, may reduce the efficacy of CDK4/6 inhibitors among Asian populations [39]. Also, further analysis is not feasible without data on sequential treatment after CDK4/6i + ET. Accordingly, the efficacy of CDK4/6i + ET should be interpreted with caution. PALOMA-2 [13] indicated an opposite outcome in the endocrine-sensitive groups. The combination of CDK4/6 inhibitors and letrozole did not show significant effects on prolonging OS, possibly due to high mortality and attrition rates observed. According to the test for subgroup differences, no significant difference (*p* = 0.30) was observed in OS between the combination of CDK4/6 + aromatase inhibitors and CDK4/6 + Fulvestrant.

Based on the findings above, this meta-analysis comprehensively explores the efficacy and safety of CDK4/6 inhibitors and discusses differences between clinical trials and our findings. However, several limitations should be acknowledged. First, the proportion of Asian participants differed across trials. PALOMA-1 [12] did not include Asians, PALOMA-4 [14] focused only on Asians, and other trials included only a few. This variation may influence the outcomes in Asian populations. Second, ER and PR positivity rates were not reported in detail. According to the protocol [12–22], patients are eligible to join the trials if breast cancer was classified as HR(+), defined as having more than 1% of cells staining either ER or PR positive. However, the clinical trials lacked detailed information on the percentage of ER and PR positivity. This limitation restricts more granular subgroup analyses. Third, data on breast cancer metastatic sites were not available, restricting the analyses of outcomes based on metastatic profile. Lastly, a new generation of CDK4/6 inhibitors has been released. However, the articles on these new drugs fall outside the inclusion deadlines set for this study. Only a few studies are available currently, and the results do not show notable differences from ours [40,41]. It is more appropriate to conduct a meta-analysis with strong evidence once more research about these new drugs becomes available. Overall, future research solving these gaps could enhance clinical recommendations for managing HR(+)/HER2(−) metastatic breast cancer.

## Conclusion

This meta-analysis shows the superior efficiency of CDK4/6 inhibitors combined with endocrine therapy compared to endocrine therapy alone in the treatment of HR(+)/HER2(−) metastatic breast cancer. Key advantages include significant improvement in progression-free survival, overall survival, objective response rate, clinical benefit rate, and a reduced rate of progress disease. CDK4/6 inhibitors are associated with adverse events, particularly hematological toxicities, similar to those seen with chemotherapy. However, these effects are generally reversible, manageable, and well-tolerated. Clinicians should be aware of hematological toxicities, liver function abnormalities, and venous thromboembolism when using CDK4/6 inhibitors. Our findings indicate that CDK4/6 inhibitors are pivotal components of treatment for HR(+)/HER2(−) metastatic breast cancer, offering patients substantial clinical benefits with acceptable toxicity profiles.

## Supplementary Material

Supplementary materials_20250621.docx

The PRISMA 2020_checklist 20250621.docx

## Data Availability

The data that support the findings of this study are available from the corresponding authors, but restrictions apply to the availability of these data upon reasonable requests.
