# Supplementary material for: Treatment consequence and adverse events of cyclin-dependent kinase 4/6 inhibitors on patients with hormone receptor-positive, HER2-negative metastatic breast cancer: a systematic review and meta-analysis
Source: Ann Med. 2025 Sep 8;57(1):2557509. doi: 10.1080/07853890.2025.2557509 (PMC12422056; doi:10.1080/07853890.2025.2557509)

**Treatment consequence and adverse events of cyclin-dependent kinase 4/6 inhibitors on patients with hormone receptor-positive, HER2-negative metastatic breast cancer: A systematic review and meta-analysis**

**Content**

[Supplementary Table 1. Summary of Sensitivity Analysis 3](#_Toc200664864)

[Supplementary Figure 1. Forest plot of the comparison of adverse events 4](#_Toc200664865)

[Supplementary Figure 1a. Forest plot of the comparison of adverse events_Neutropenia 4](#_Toc200664866)

[Supplementary Figure 1b. Forest plot of the comparison of adverse events_Leukopenia 4](#_Toc200664867)

[Supplementary Figure 1c. Forest plot of the comparison of adverse events_ Anemia 4](#_Toc200664868)

[Supplementary Figure 1d. Forest plot of the comparison of adverse events_ Thrombocytopenia 4](#_Toc200664869)

[Supplementary Figure 1e. Forest plot of the comparison of adverse events_ Diarrhea 5](#_Toc200664870)

[Supplementary Figure 1f. Forest plot of the comparison of adverse events_ Nausea 5](#_Toc200664871)

[Supplementary Figure 1g. Forest plot of the comparison of adverse events_ Fatigue 5](#_Toc200664872)

[Supplementary Figure 1h. Forest plot of the comparison of adverse events_ Abnormal LFTse 5](#_Toc200664873)

[Supplementary Figure 1i. Forest plot of the comparison of adverse events_ Vomiting 6](#_Toc200664874)

[Supplementary Figure 1j. Forest plot of the comparison of adverse events_ Arthralgia 6](#_Toc200664875)

[Supplementary Figure 1k. Forest plot of the comparison of adverse events_ Alopecia 6](#_Toc200664876)

[Supplementary Figure 1l. Forest plot of the comparison of adverse events_ Decreased appetite 6](#_Toc200664877)

[Supplementary Figure 1m. Forest plot of the comparison of adverse events_ QT prolong 7](#_Toc200664878)

[Supplementary Figure 1n. Forest plot of the comparison of severe adverse events_ Venous thromboembolism (VTE) 7](#_Toc200664879)

[Supplementary Figure 2. Subgroup analysis 8](#_Toc200664880)

[Supplementary Figure 2a. Subgroup analysis of progression-free survival_Efficacy in First-Line vs. Efficacy in Second-Line 8](#_Toc200664881)

[Supplementary Figure 2b. Subgroup analysis of progression-free survival_Visceral metastases vs. Non-visceral metastases 8](#_Toc200664882)

[Supplementary Figure 2c. Subgroup analysis of progression-free survival_Bone only metastases vs. Non-Bone only metastases 9](#_Toc200664883)

[Supplementary Figure 2d. Subgroup analysis of progression-free survival_Asian vs. Non-Asian 9](#_Toc200664884)

[Supplementary Figure 2e. Subgroup analysis of progression-free survival_ER and PR positive vs. Other 10](#_Toc200664885)

[Supplementary Figure 2f. Subgroup analysis of progression-free survival_<21 years old vs.≧21 years old 10](#_Toc200664886)

[Supplementary Figure 2g. Subgroup analysis of progression-free survival_ECOG:0 vs. ECOG≧1 11](#_Toc200664887)

[Supplementary Figure 2h. Subgroup analysis of progression-free survival_Chemotherapy for (neo)adjuvant therapy vs. no prior chemotherapy 11](#_Toc200664888)

[Supplementary Figure 2i. Subgroup analysis of progression-free survival_Number of organs involved (1 vs. 2 vs. 1) 12](#_Toc200664889)

[Supplementary Figure 2j. Subgroup analysis of progression-free survival_Post-menopausal vs. Pre/Peri-menopausal 12](#_Toc200664890)

[Supplementary Figure 2k. Subgroup analysis of progression-free survival_Endocrine status 13](#_Toc200664891)

[Supplementary Figure 2l. Subgroup analysis of progression-free survival_Endocrine Therapy Medication 14](#_Toc200664892)

[Supplementary Figure 2m. Subgroup analysis of overall survival_Endocrine Therapy Medication 15](#_Toc200664893)

[Supplementary Figure 3. Funnel Plot of Progression-Free Survival 15](#_Toc200664894)

[Supplementary Figure 4. Funnel Plot of Overall Survival 17](#_Toc200664895)

[Supplementary Figure 5. Funnel Plot of Objective Response Rate 18](#_Toc200664896)

[Supplementary Figure 5a. Funnel Plot of Objective Response Rate in intention-to-treat analysis 18](#_Toc200664897)

[Supplementary Figure 5b. Funnel Plot of Objective Response Rate in measurable disease analysis 18](#_Toc200664898)

[Supplementary Figure 6. Funnel Plot of Clinical Benefit Rate 19](#_Toc200664899)

[Supplementary Figure 6a. Funnel Plot of Clinical Benefit Rate in intention-to-treat analysis 19](#_Toc200664900)

[Supplementary Figure 6b. Funnel Plot of Clinical Benefit Rate in measurable disease analysis 19](#_Toc200664901)

# Supplementary Table 1. Summary of Sensitivity Analysis

| Subgroup Analysis | Heterogeneity (I-square)  Before exclusion | Exclude Trials | Heterogeneity (I-square)  After exclusion |
| --- | --- | --- | --- |
| Bone only metastasis | 36% | FLIPPER | 10%(HR:0.50,0.40-0.62) |
| Asian | 45% | MONALEESA-3 | 27%(HR:0.47,0.3-0.59) |
| Non-Asian | 34% | MONARCH PLUS | 21%(HR:0.57,0.51-0.64) |
| ORR  (ITT) | 53% | MONARCH-2  MONARCH PLUS | 40%(RR:1.45,1.29-1.62)  35%(RR:1.44,1.29-1.61) |
| ORR  (measurable disease) | 58% | MONARCH-2  MONARCH PLUS | 43%(RR:1.45,1.34-1.57)  46%(RR:1.46,1.34-1.58) |
| CBR  (ITT) | 74% | PALOMA-3  MONARCH PLUS | 60%(RR:1.15,1.08-1.22)  70%(RR:1.16,1.08-1.24) |
| CBR  (measurable disease) | 65% | PALOMA-3  PALOMA-4 | 46%(RR:1.16,1.10-1.23)  50%(RR:1.22,1.14-1.30) |

# Supplementary Figure 1. Forest plot of the comparison of adverse events

## Supplementary Figure 1a. Forest plot of the comparison of adverse events_Neutropenia


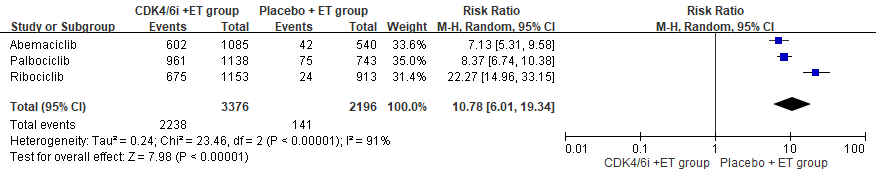


## Supplementary Figure 1b. Forest plot of the comparison of adverse events_Leukopenia


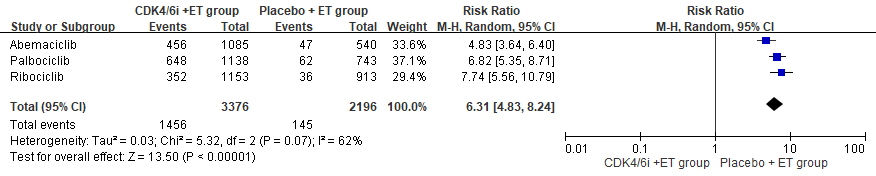


## Supplementary Figure 1c. Forest plot of the comparison of adverse events_ Anemia


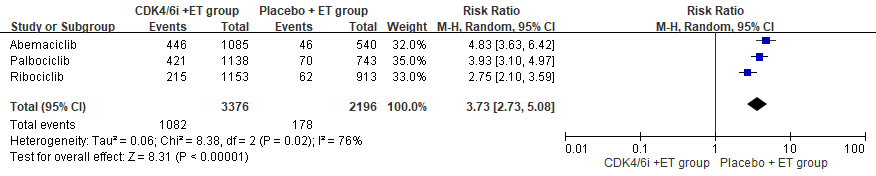


## Supplementary Figure 1d. Forest plot of the comparison of adverse events_ Thrombocytopenia


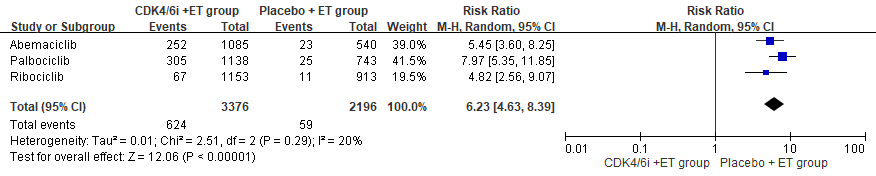


## Supplementary Figure 1e. Forest plot of the comparison of adverse events_ Diarrhea


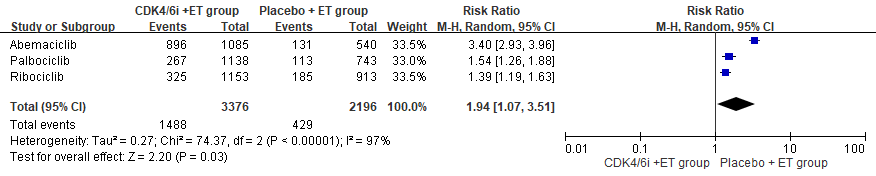


## Supplementary Figure 1f. Forest plot of the comparison of adverse events_ Nausea


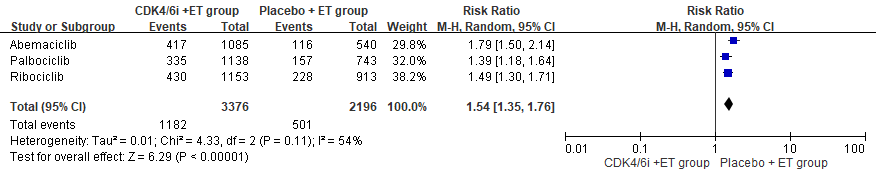


## Supplementary Figure 1g. Forest plot of the comparison of adverse events_ Fatigue


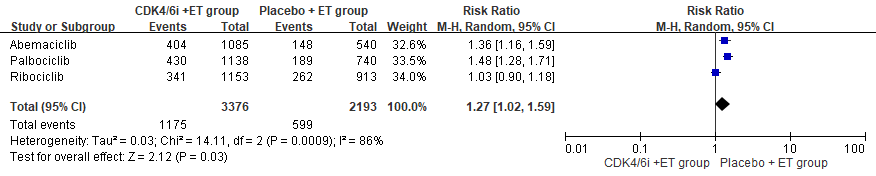


## Supplementary Figure 1h. Forest plot of the comparison of adverse events_ Abnormal LFTse


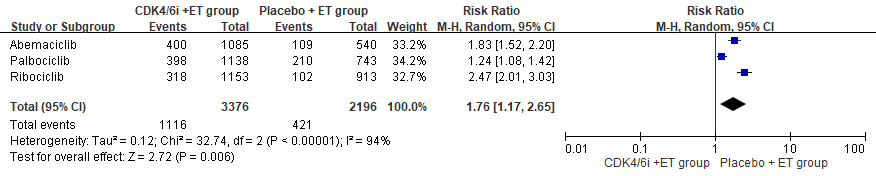


## Supplementary Figure 1i. Forest plot of the comparison of adverse events_ Vomiting


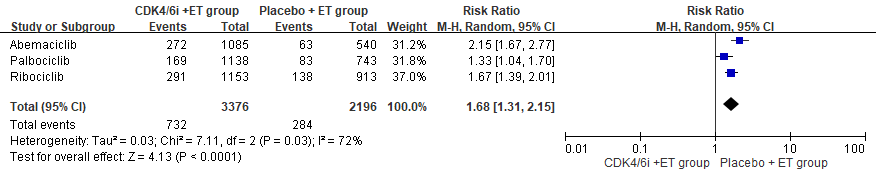


## Supplementary Figure 1j. Forest plot of the comparison of adverse events_ Arthralgia


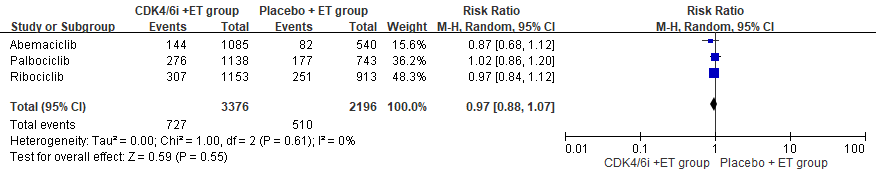


## Supplementary Figure 1k. Forest plot of the comparison of adverse events_ Alopecia


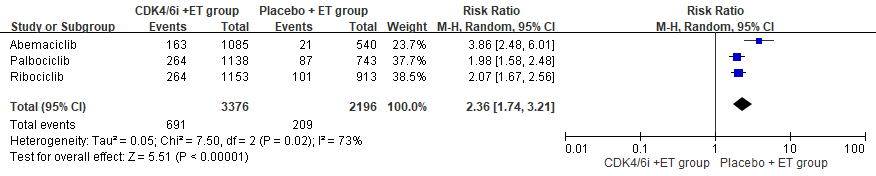


## Supplementary Figure 1l. Forest plot of the comparison of adverse events_ Decreased appetite


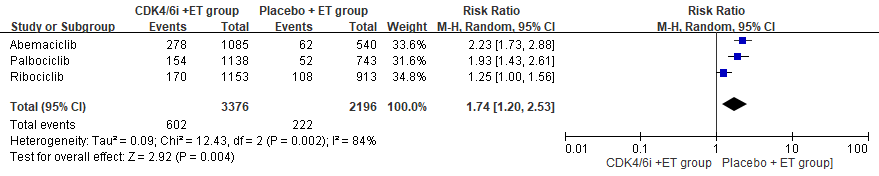


## Supplementary Figure 1m. Forest plot of the comparison of adverse events_ QT prolong


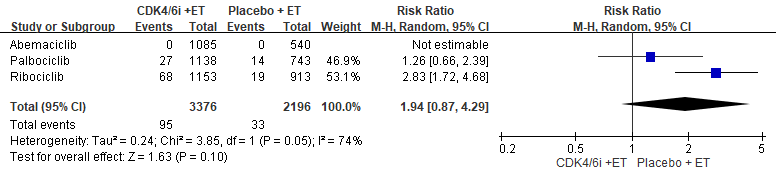


## Supplementary Figure 1n. Forest plot of the comparison of severe adverse events_ Venous thromboembolism (VTE)


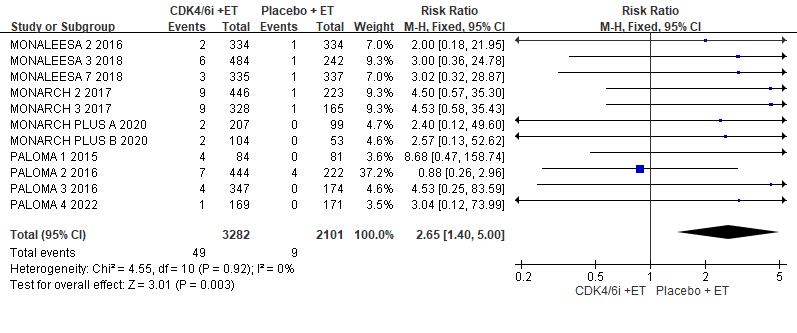


# Supplementary Figure 2. Subgroup analysis

## Supplementary Figure 2a. Subgroup analysis of progression-free survival_Efficacy in First-Line vs. Efficacy in Second-Line


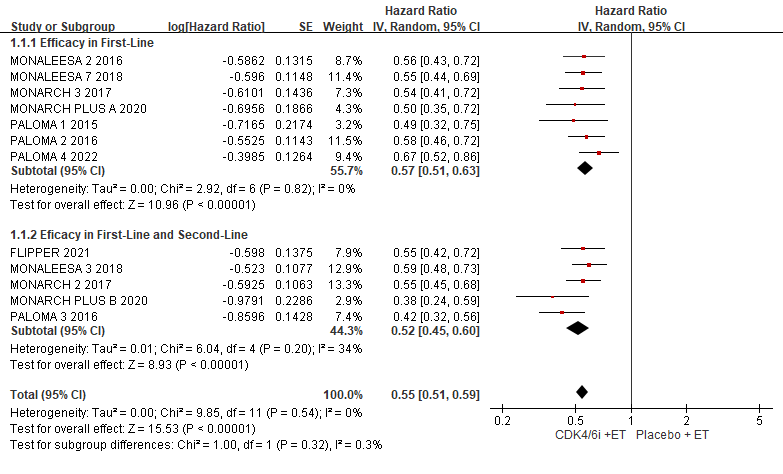


## Supplementary Figure 2b. Subgroup analysis of progression-free survival_Visceral metastases vs. Non-visceral metastases


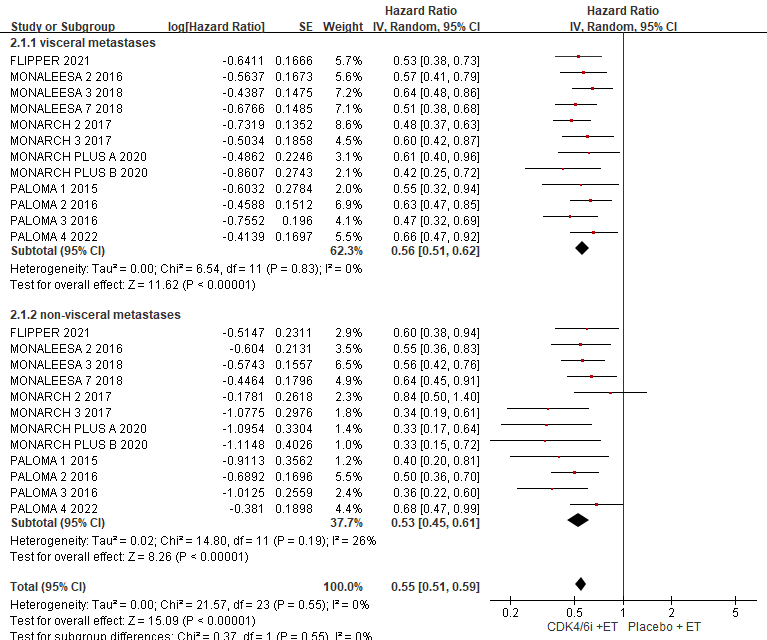


##
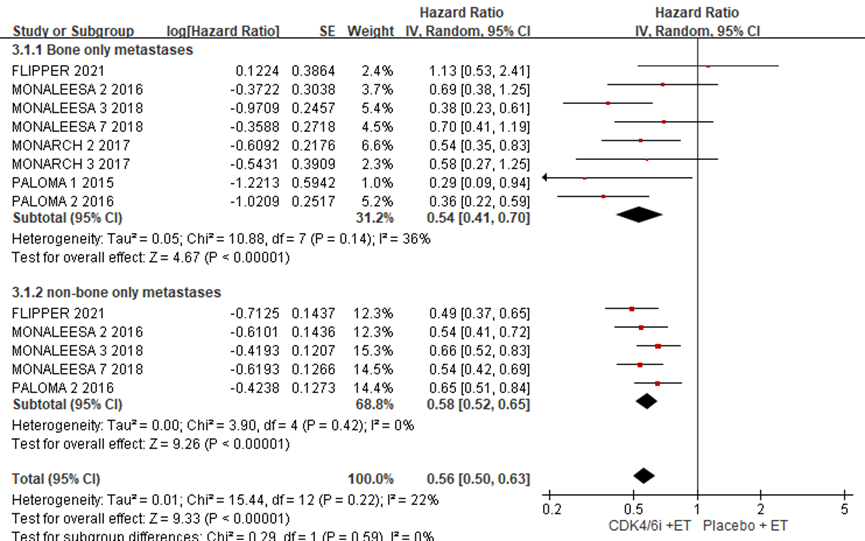
Supplementary Figure 2c. Subgroup analysis of progression-free survival_Bone only metastases vs. Non-Bone only metastases

##
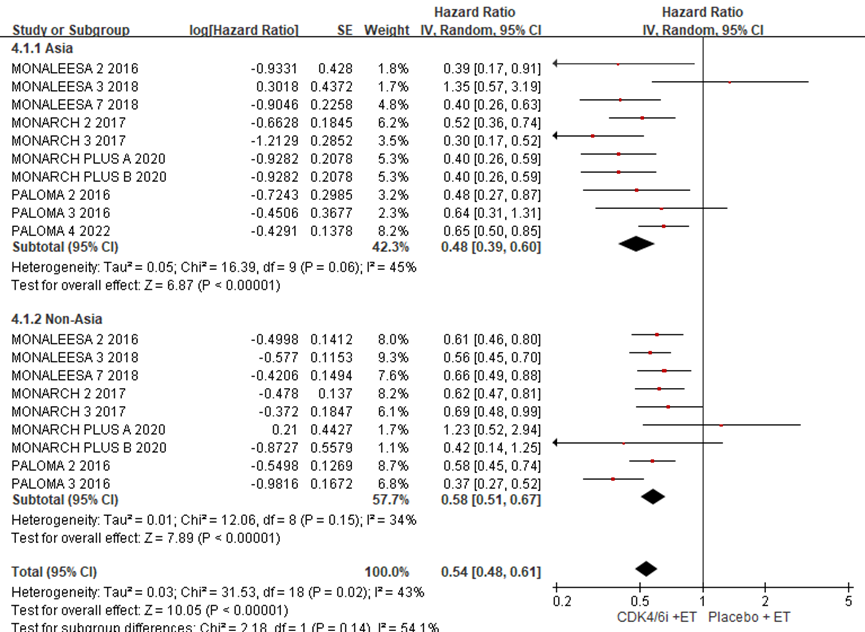
Supplementary Figure 2d. Subgroup analysis of progression-free survival_Asian vs. Non-Asian

## Supplementary Figure 2e. Subgroup analysis of progression-free survival_ER and PR positive vs. Other


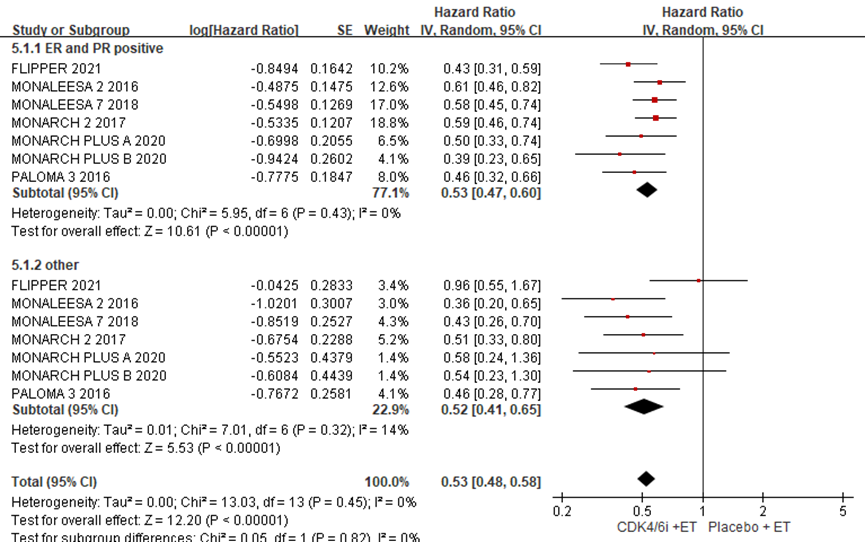


## Supplementary Figure 2f. Subgroup analysis of progression-free survival_<65 years old vs.≧65 years old


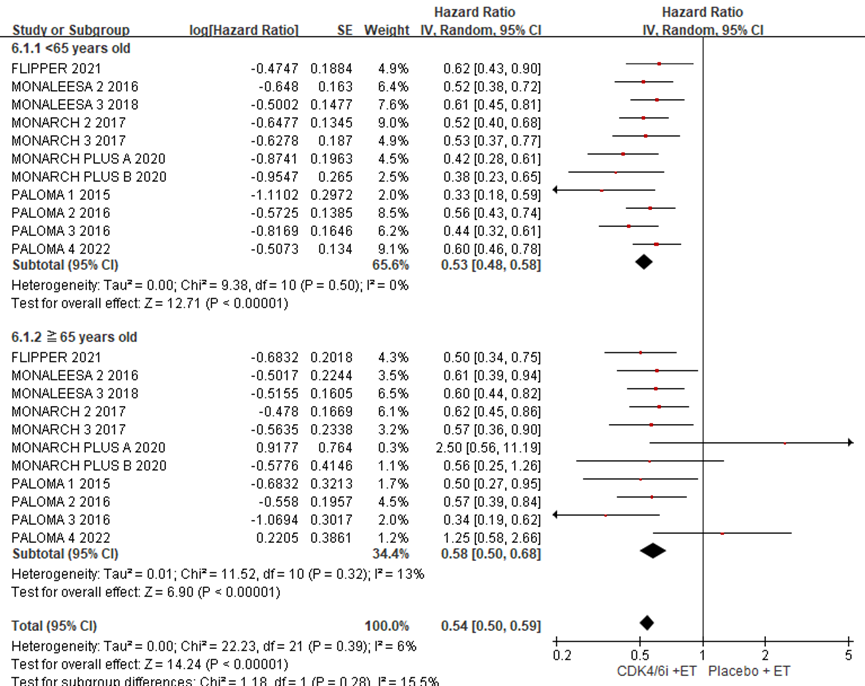


##
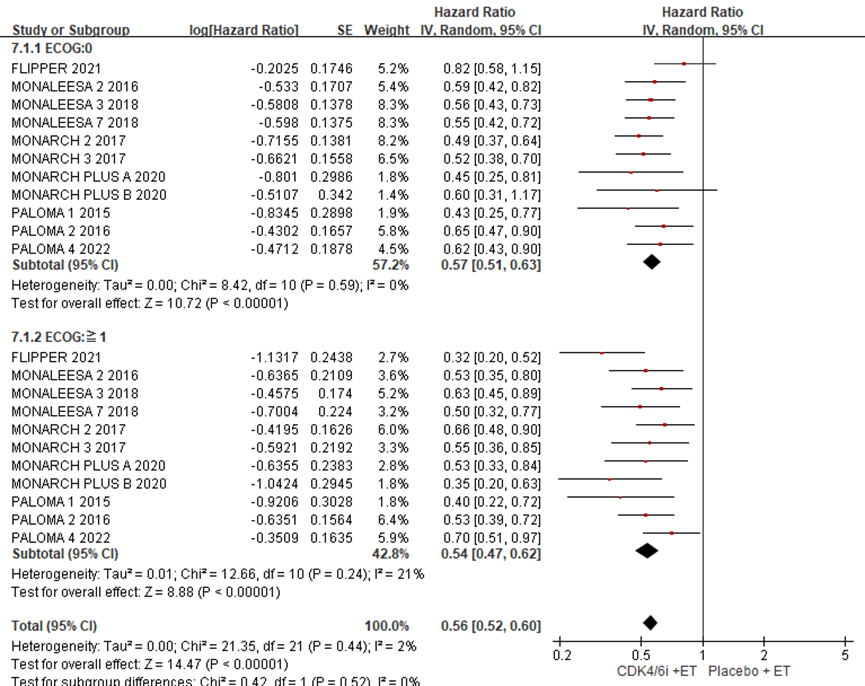
Supplementary Figure 2g. Subgroup analysis of progression-free survival_ECOG:0 vs. ECOG≧1

## Supplementary Figure 2h. Subgroup analysis of progression-free survival_Chemotherapy for (neo)adjuvant therapy vs. no prior chemotherapy


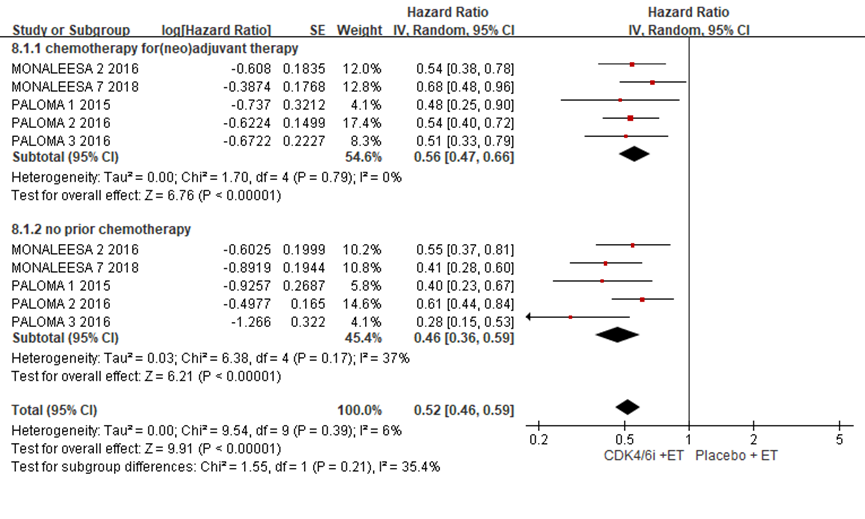


##
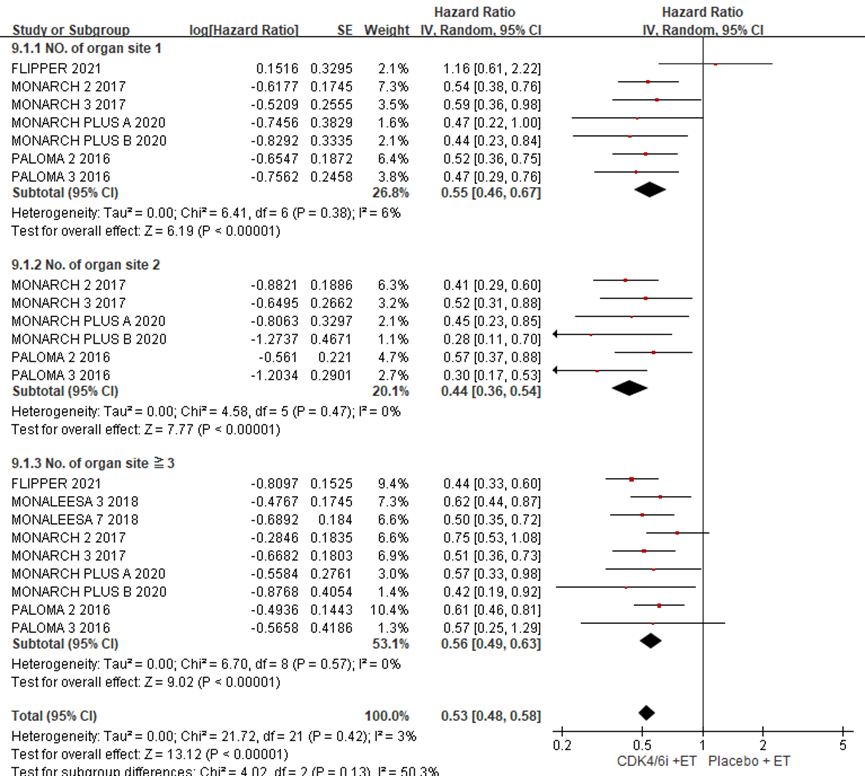
Supplementary Figure 2i. Subgroup analysis of progression-free survival_Number of organs involved (1 vs. 2 vs. 1)

## Supplementary Figure 2j. Subgroup analysis of progression-free survival_Post-menopausal vs. Pre/Peri-menopausal


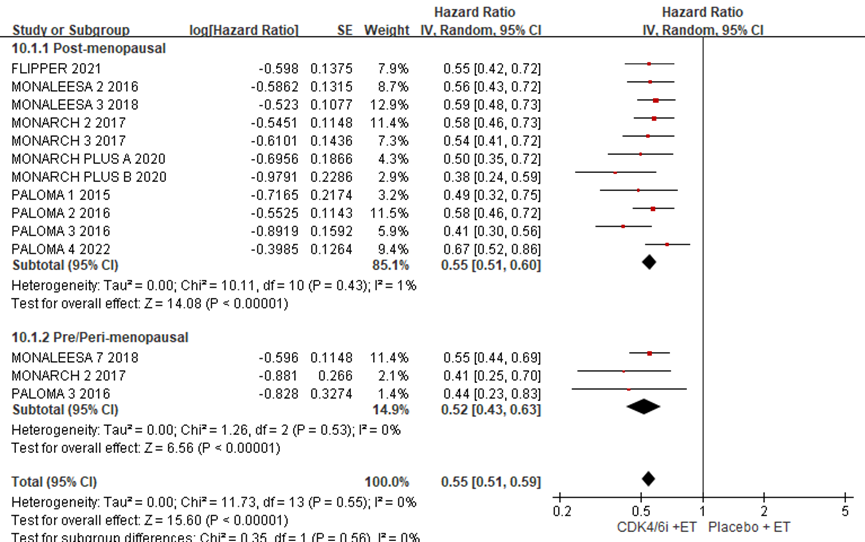


##
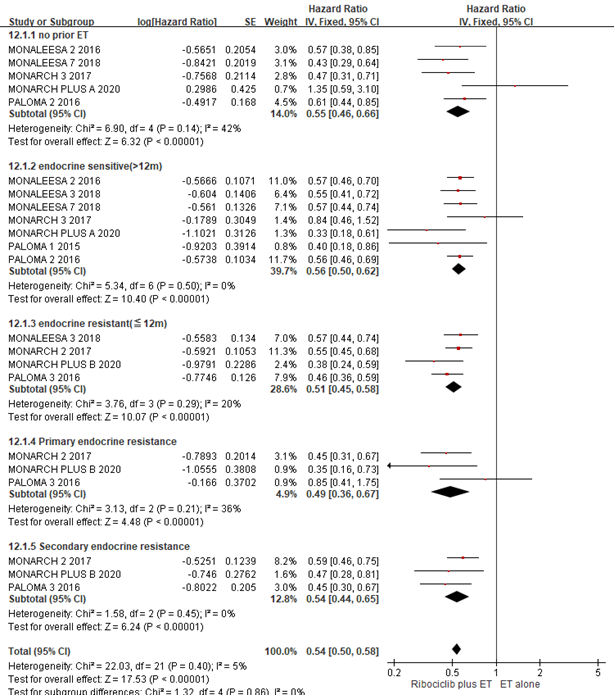
Supplementary Figure 2k. Subgroup analysis of progression-free survival_Endocrine status

##
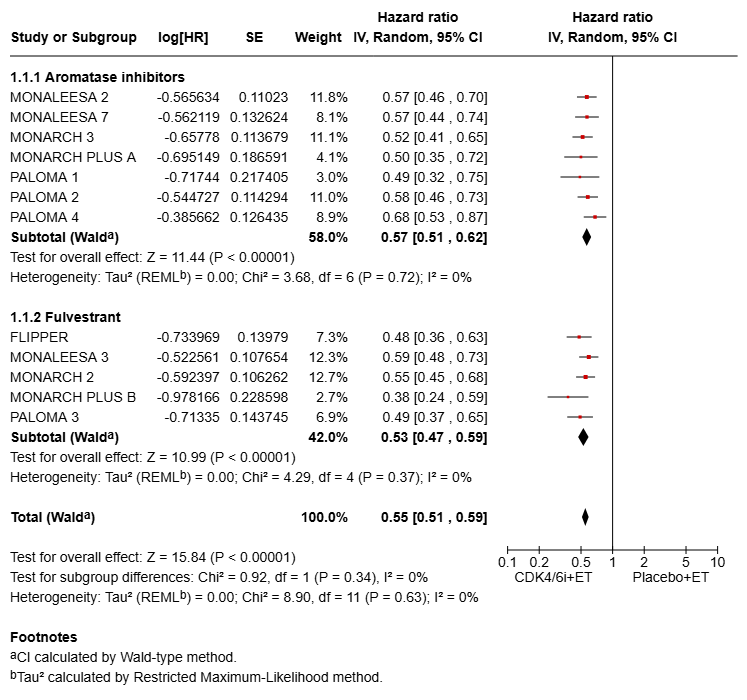
Supplementary Figure 2l. Subgroup analysis of progression-free survival_Endocrine Therapy Medication

##
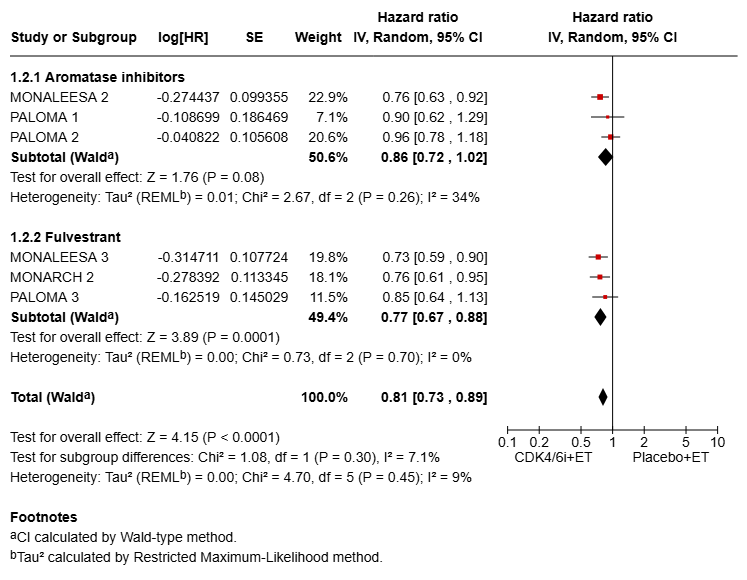
Supplementary Figure 2m. Subgroup analysis of overall survival_Endocrine Therapy Medication

# Supplementary Figure 3. Funnel Plot of Progression-Free Survival


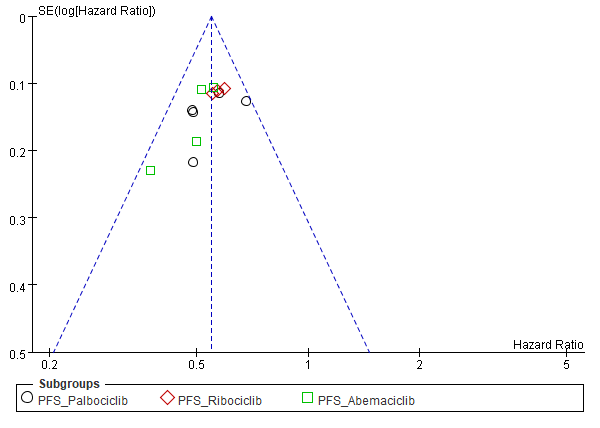


# Supplementary Figure 4. Funnel Plot of Overall Survival


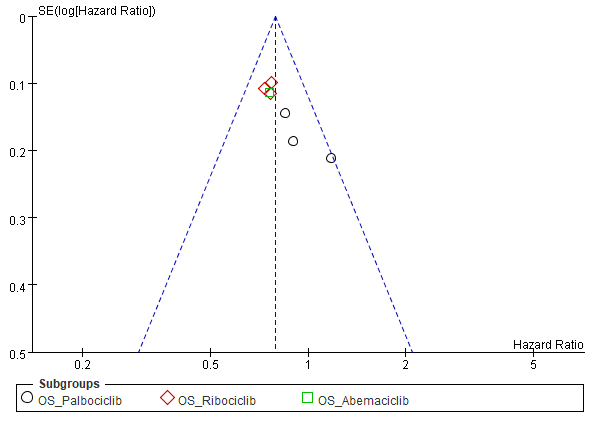


# Supplementary Figure 5. Funnel Plot of Objective Response Rate

## Supplementary Figure 5a. Funnel Plot of Objective Response Rate in intention-to-treat analysis


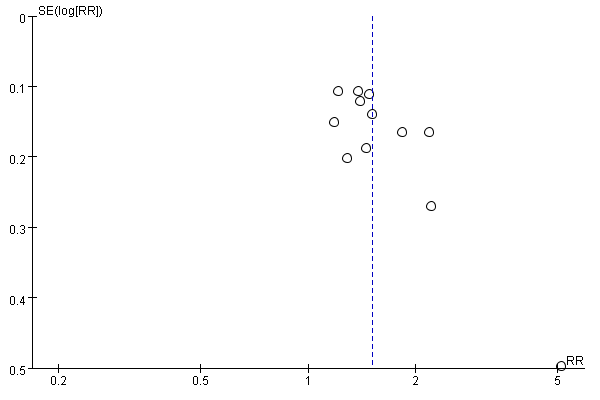


## Supplementary Figure 5b. Funnel Plot of Objective Response Rate in measurable disease analysis


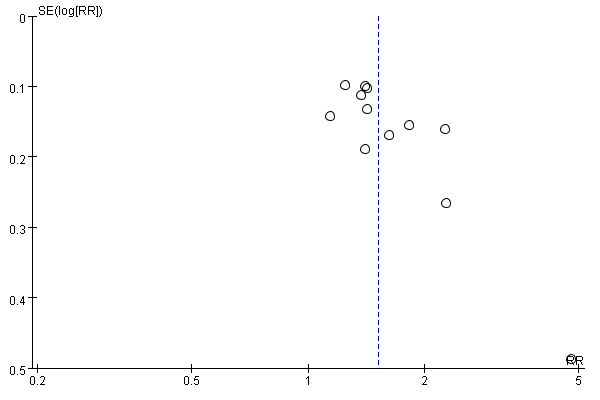


# Supplementary Figure 6. Funnel Plot of Clinical Benefit Rate

Supplementary Figure 6a. Funnel Plot of Clinical Benefit Rate in intention-to-treat analysis
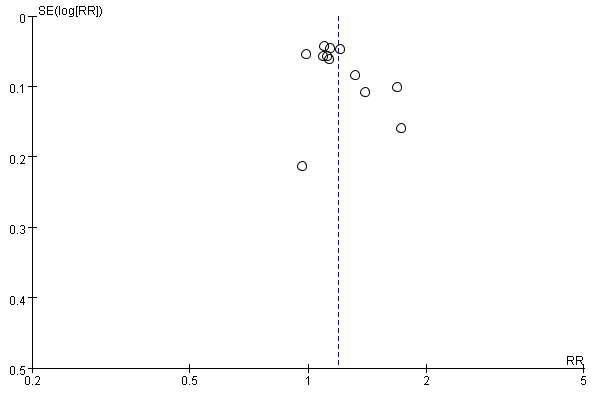


## Supplementary Figure 6b. Funnel Plot of Clinical Benefit Rate in measurable disease analysis


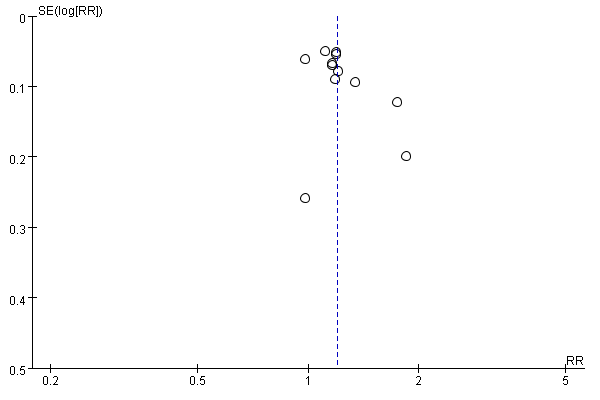

Supplement: Supplementary materials_20250621.docx [file IANN_A_2557509_SM3088.docx]
